# Conditioned Place Avoidance of Zebrafish (*Danio rerio*) to Three Chemicals Used for Euthanasia and Anaesthesia

**DOI:** 10.1371/journal.pone.0088030

**Published:** 2014-02-04

**Authors:** Devina Wong, Marina A. G. von Keyserlingk, Jeffrey G. Richards, Daniel M. Weary

**Affiliations:** 1 Animal Welfare Program, University of British Columbia, Vancouver, British Columbia, Canada; 2 Department of Zoology, University of British Columbia, Vancouver, British Columbia, Canada; Tulane University Medical School, United States of America

## Abstract

Zebrafish are becoming one of the most used vertebrates in developmental and biomedical research. Fish are commonly killed at the end of an experiment with an overdose of tricaine methanesulfonate (TMS, also known as MS-222), but to date little research has assessed if exposure to this or other agents qualifies as euthanasia (i.e. a “good death”). Alternative agents include metomidate hydrochloride and clove oil. We use a conditioned place avoidance paradigm to compare aversion to TMS, clove oil, and metomidate hydrochloride. Zebrafish (n = 51) were exposed to the different anaesthetics in the initially preferred side of a light/dark box. After exposure to TMS zebrafish spent less time in their previously preferred side; aversion was less pronounced following exposure to metomidate hydrochloride and clove oil. Nine of 17 fish exposed to TMS chose not to re-enter the previously preferred side, versus 2 of 18 and 3 of 16 refusals for metomidate hydrochloride and clove oil, respectively. We conclude that metomidate hydrochloride and clove oil are less aversive than TMS and that these agents be used as humane alternatives to TMS for killing zebrafish.

## Introduction

Zebrafish (*Danio rerio*) are a popular vertebrate model for developmental studies and biomedical research [1], [2]. In 2010, over half the animals used in Canada for scientific purposes were fish [3]. With rapid increases in fish use more research is required on the welfare of these animals, including how to minimize the pain and distress caused by experimental procedures. The most common of these procedures is euthanasia. Almost every fish used in research is euthanized at the end of the study with an overdose of anaesthetic [4], [5]. Fish are anaesthetized via immersion in water containing an agent such as the widely used tricaine methanesulfonate (TMS; also known as MS-222) [6], [7]. TMS and metomidate hydrochloride (metomidate; also known as Aquacalm™) are the two agents approved for veterinary use in Canada. Clove oil (approximately 95% eugenol) is an alternative agent considered by some researchers to be equally or more effective than TMS as a fish anaesthetic [8], [9]. Clove oil shares the same main active ingredient as that in approved fish anaesthetics in several countries including Australia, Chile and Norway [6].

Research into humane euthanasia methods in rodents has made use of a light/dark shuttle box [10], commonly used to assess anxiety [11]–[13]. There are however conflicting reports on zebrafish preferences for light or dark environments. For example, Steenbergen et al. [14] found that adult zebrafish prefer light, but other work has found a preference for dark [15]. Conditioned place avoidance is a behavioural paradigm that has been used to determine reinforcing effects of aversive experiences [16]. The aversive experience is paired with a previously neutral environment and results in avoidance of the paired environment; behavioural parameters such as number of entries and time spent in the compartment can be used to assess the degree of avoidance [1].

The aim of the current study was to use a light/dark box to test aversion-avoidance and conditioned place avoidance in response to exposure to TMS, metomidate, and clove oil. During baseline testing fish were not exposed to any agent; behaviour during this stage was used to establish the preferred location (light or dark side of the tank) for each fish. We used light/dark environments (instead of the more typically used neutral environments) to take advantage of the natural preference and provide a stronger test of conditioned place aversion (by switching preferences, rather than just establishing a preference). Each individual was then tested with one anaesthetic added to the preferred compartment. We predicted that when tested subsequently, with no anaesthetic present in the tank, the fish would avoid the previously preferred compartment.

## Materials and Methods

### Ethics statement

This protocol was approved by the University of British Columbia Animal Care Committee (application number: A11-0252). All experimental procedures were performed in accordance with the Canadian Council on Animal Care guidelines on care and use of fish in research.

### Animals and housing

Fifty-four 6-month old zebrafish (mixed sex, mean±s.d. weight 0.38±0.05 g) were obtained from a collaborating research laboratory that had originally purchased the fish from a local pet store (Noah's Pet Ark, Vancouver, BC). Fish were housed in two groups of 27, in two 12.0 L, 300×195×205 mm acrylic tanks (Faunarium Plastic Terrarium, ExoTerra PT-2260, Montreal, Canada) and fed once a day with fish flakes until satiation (Nutrafin Max Tropical Fish Flakes, Montreal, Canada). Water was filtered using mechanical (sponge) and chemical (activated carbon) filtration units (AquaClear Power Filter, Montreal, Canada). The waterfall design of the filtration unit also allowed water to be thoroughly aerated. Approximately 15% of the tank water was changed every week. To ensure de-chlorination and removal of toxic metals, tap water was mixed with a de-chlorinator and de-metalizer (Nutrafin® Aqua Plus Tap Water Conditioner, West Yorkshire, UK) and allowed to sit for at least 24 h before use. Seachem Equilibruim™ (Seachem Laboratories, Inc., Madison, GA, USA) was added to raise the hardness of the water to at least 80 ppm, and Alkaline Buffer™ (Seachem Laboratories, Inc., Madison, GA, USA) was added to maintain hardness. The water temperature was maintained at 26°C(±2°C) and lights were on between 0700 h and 1900 h. All testing and feeding were conducted during the light phase between 1100 h and 1500 h.

### Experimental apparatus

**Figure 1:**
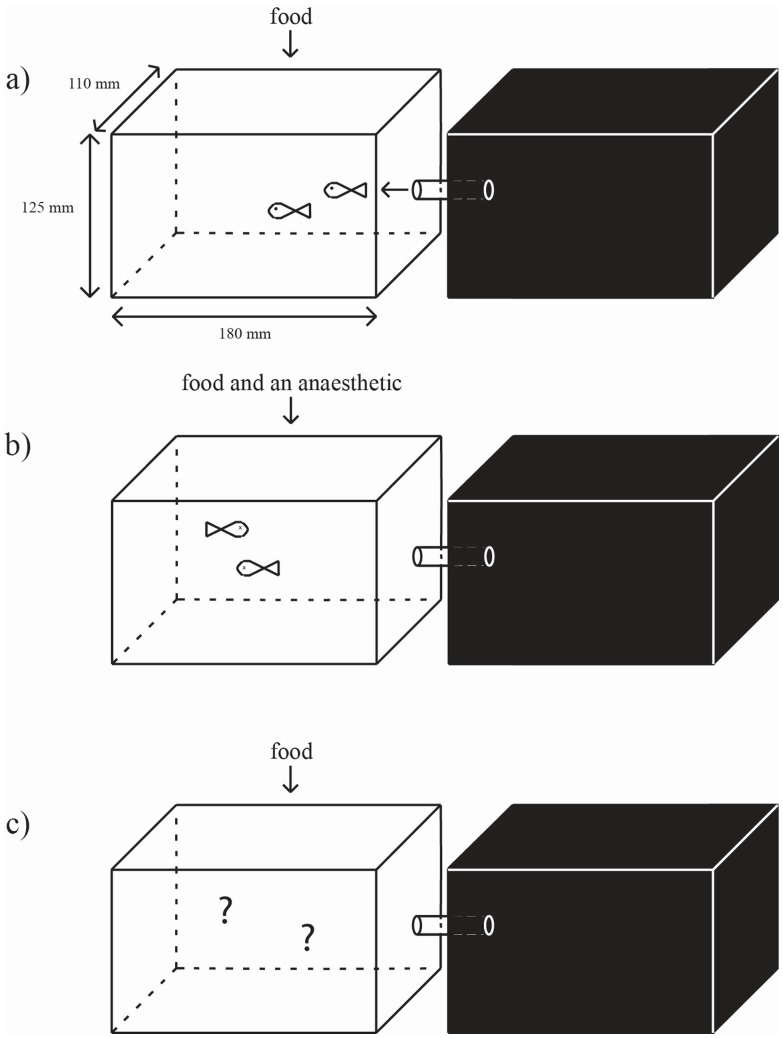
Diagram of testing apparatus. The testing apparatus, consisting of a light and dark side identical in size. During the initial preference determination period (a) we simply monitored the time spent on the two-sides of the tank (all fish tested preferred the light side). Fish were then exposed to one of three anaesthetics while in the light side of the tank (b). Once fish recovered we re-tested preference (following the identical procedure as used in panel a) with the apparatus once again free of anaesthetics (c).

During training and testing fish were housed in pairs in the testing apparatus consisting of two 2.5 L acrylic tanks (Faunarium Plastic Terrarium, ExoTerra PT-2250, Montreal, Canada) connected with a 5.0-cm long, 2.8-cm diameter PVC tube (Figure 1). Preliminary testing with dyed water in one of the two tanks showed minimal diffusion; after 24 h the dye in the water did not extend beyond the half way mark of the connecting tube.

By keeping fish in pairs we were able to still visually identify individuals and provide some social stimulus. Plastic plants were used to create hiding spaces and mitigate dominance-related aggression behaviours. The sides of both tanks were covered with black, light-proof plastic but one side had a light-proof lid and the other side was lit with a 40 W incandescent bulb. Water temperature was kept at 26±2°C and both sides were aerated with an air stone.

A number of studies show zebrafish to be phototaxic (light preference [14], [17], [18]; dark preference [1], [19]) so fish were initially introduced into the light side of the apparatus. Fish were allowed to habituate to the apparatus for 48 h before testing. The training period lasted between 3 to 10 days.

### Solution preparation

To prepare solutions, clove oil (95% eugenol, Sigma-Aldrich, USA) was dissolved in 95% ethanol at a 1∶9 ratio (clove oil:ethanol) and then further diluted with tank holding water (following [20]). The resulting mixture had a pH between 6.5 – 7.0. Buffered TMS solution was prepared by dissolving ethyl 3-aminobenzoate methanesulfonate powder (Sigma-Aldrich, USA) into a sample of the tank holding water and then buffering the solution with sodium bicarbonate until pH reached 7.0 (following [21]). Metomidate was prepared by dissolving Aquacalm™ directly into a sample of tank holding water. The resulting pH was 7.0; no buffering was required. Temperatures of these solutions were equilibrated to that of the test tank. Aliquots of the stock solution were then used to achieve the desired concentrations in the test tank.

Concentrations of TMS, metomidate, and clove oil used to achieve anaesthesia vary widely in the literature (TMS: [21]–[25]; metomidate: [21], [24], [25]; clove oil: [26]–[28]). To allow direct comparisons between the three anaesthetics, equipotent concentrations were used. In preliminary testing we exposed 10 zebrafish (these fish were not used in the main study) to TMS at 100, 150, 200 mg/L, (approximate ranges used in previous studies), clove oil at 45, 55, 65 mg/L and metomidate at 8, 12, 15 mg/L (reflecting the range of values reported in the literature). Each fish was exposed to one of nine possible anaesthetic and concentration combinations, and allowed 24 h recovery before being exposed to the next combination. Order was randomized. We measured the latency until the fish were unconscious (defined as loss of equilibrium in the water column and loss of reflex, defined as lack of reaction to being netted). Graphical interpolation was used to obtain doses for each agent that achieved loss of equilibrium at approximately 75 s; this resulted in a concentration of 150 mg/L for TMS, 55 mg/L for clove oil and 13.5 mg/L for metomidate (Table 1).

**Table 1:** Mean and standard deviation for test doses of the three euthanasia agents.

	Low	Medium	High
TMS	177.7±45.1	75.3±6.7	60.0±8.9
Clove oil	122.4±22.1	74.9±10.2	50.9±4.7
Metomidate hydrochloride	119.8±24.1	87.0±12.0	65.2±10.7

Mean±S.D. times to loss of equilibrium for three doses of TMS (n = 10), clove oil (n = 10), and metomidate hydrochloride (n = 10). These values were used to determine equipotent concentrations by graphical interpolation.

### Training and testing

Before testing, fish were trained to cross through the PVC tube from one side of the apparatus to the other. Training occurred at feeding time to reinforce preference. The condition that the fish were found in before conditions were switched was deemed the ‘preferred side’. Out of the 54 fish tested 53 showed an initial preference for the light side. The one fish that preferred the dark side was removed and replaced with a fish that also preferred the light side and acted as a pair-mate but was not included in the analysis.

**Figure 2:**
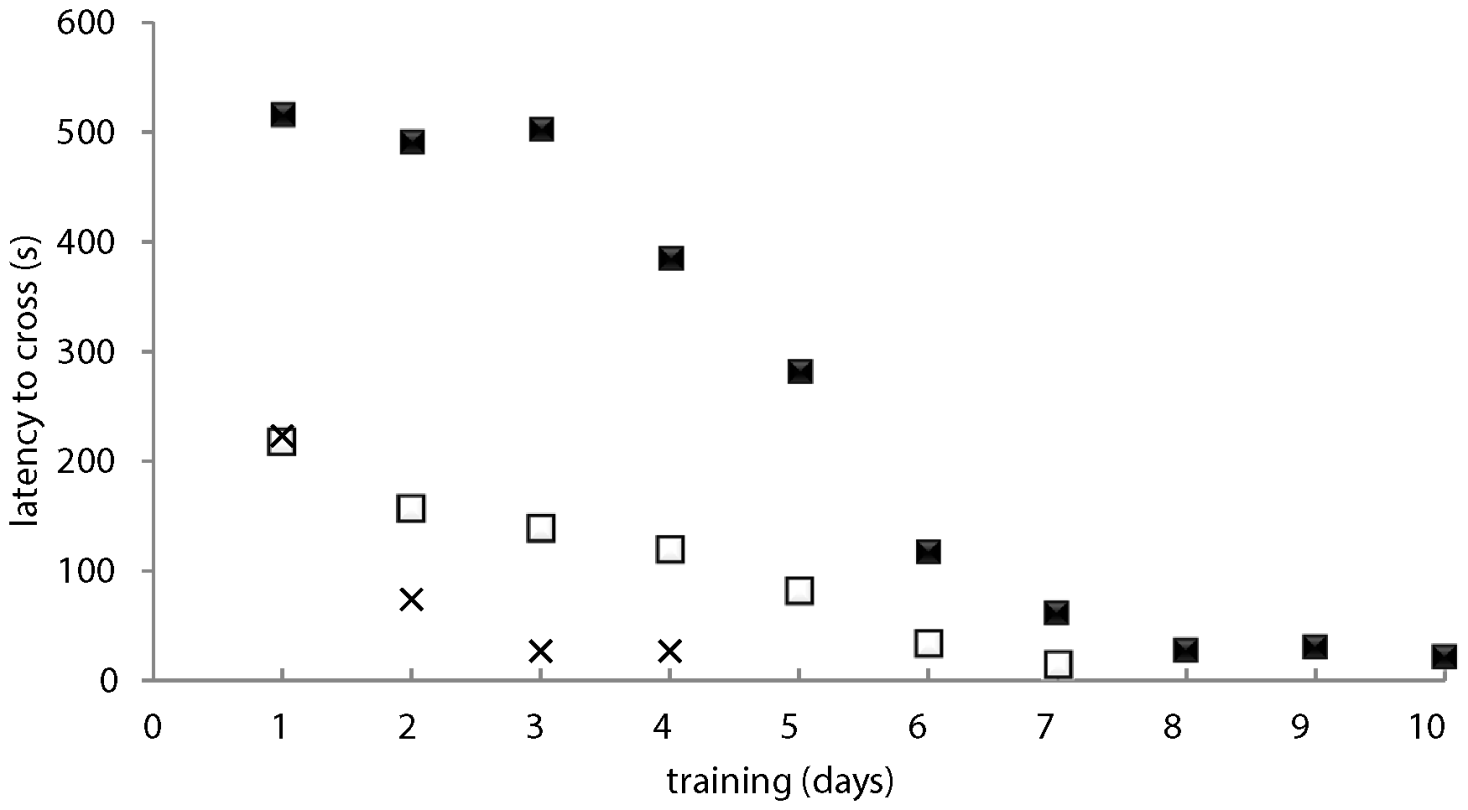
Learning curve for fish with different learning speeds. Latency for fish to cross the tunnel into the preferred over the training period. Three fish are illustrated: the fastest learner (black crosses) that reached the learning criterion in 3 d, the slowest learner that reached criterion in 10 d (black squares) and an intermediate fish (white squares) that reached criterion in 6 d.

Immediately before feeding, the light/dark conditions were switched by moving the light and switching the lid. Food was then dropped into the now lit side, prompting fish to enter. Fish were considered ready for testing once they both consistently crossed into the light side within 1 min of the switch. Training sessions occurred 5 times per day; within 10 days of training all fish met this learning criterion regardless of learning differences at the beginning of training (Figure 2).

Concurrently during training sessions fish were also habituated to our net transfer technique. Fish were trained using a food reward to voluntarily enter a net that was lined with plastic; the net was then gently lifted and used to slowly lift the fish out of one tank and lower it into another. This technique minimized any distress associated with handling and allowed fish to remain submerged throughout the transfer.

On the day of testing, time spent in each side of the apparatus before and after crossing into the preferred side was recorded for 15 min before testing to demonstrate place preference before exposure to chemicals. This phase of the experiment also served as the control, as all handling and treatment was identical to that during testing.

Twenty-seven pairs of fish were used in this experiment, nine pairs for each anaesthetic. Each fish was exposed to anaesthetic only once. Three trials were run per day, one for each anaesthetic, with the order of use determined by a 3×3 Latin square. At the beginning of the trial, a divider was used to block the tunnel entrance where the fish were in the preferred side and the anaesthetic was mixed thoroughly in the non-preferred side. The divider was then removed, and as in the training sessions, the light/dark conditions were switched and food was added signalling that the fish could enter. Trials ended when fish lost equilibrium in the water column. As soon as a fish lost equilibrium it was removed (to avoid overdose), regardless of whether their pair-mate had also lost equilibrium. Doses were selected so that fish lost equilibrium in the water column within 3 min. If fish left the anaesthetic chamber before loss of equilibrium the trial was allowed to continue for 15 min.

Fish generally recover fully from immersion anaesthetics within 5 min [9]. We kept fish in the recovery tank for 15 min to ensure complete recovery (including complete control of equilibrium). During this period the testing apparatus was cleaned first with 75% ethanol and then rinsed with fresh water. Fish were then returned to the non-preferred side of the testing apparatus with the divider in place. After 5 min the divider was again removed, and following the identical procedure used in training we recorded the time spent on each side of the apparatus for 15 min.

Each trial was video recorded from above to measure latency to cross from the dark to light side, total time spent in the light side, the number of entries into the light side, and the number of attempted entries into the light side (defined as fish coming within 1 cm of tunnel entrance, with head facing towards the tunnel, but without actually entering), and complete rejections (defined as no attempted entry into the light side). Videos were scored by an observer who blind to treatment.

### Statistical methods

The difference in the total time spent in the previously preferred side before versus after exposure was calculated for each fish (data is available as support information). The effect of anaesthetic treatment on these differences, and on the latency to cross from the non-preferred side to the preferred side during anaesthetic exposure, was tested using a Wilcoxon test. Pairwise differences between treatments in the number of complete rejections of the preferred side after anaesthetic exposure were tested using Fisher's Exact test.

The fish were tested in pairs, but we suggest that the most appropriate experimental unit for analysis was the individual as fish could and did display different responses to their pair-mate (in 11 of the 27 pairs tested). An alternative approach is to consider the pair as the experimental unit. We therefore also calculated the average difference and latency for each pair, and re-ran the Wilcoxon tests described above using the pair average as the experimental unit. The pair-based analysis resulted in identical conclusions to the individually based analysis, so only the individually based test statistics are reported. All tests were 2-tailed with alpha set at 0.05.

## Results

During exposure testing two fish never entered the chemical compartment (one for TMS and one for clove oil). Of the remaining 51 fish, 50 entered the chemical compartment and lost equilibrium in the water column without leaving. The one remaining fish left the clove oil after 3 s, but re-entered after 16 s and stayed until loss of equilibrium. There was no effect of anaesthetic treatment on latency to enter the test compartment (mode 0 s, median 17 s, inter-quartile range 181 s).

**Figure 3:**
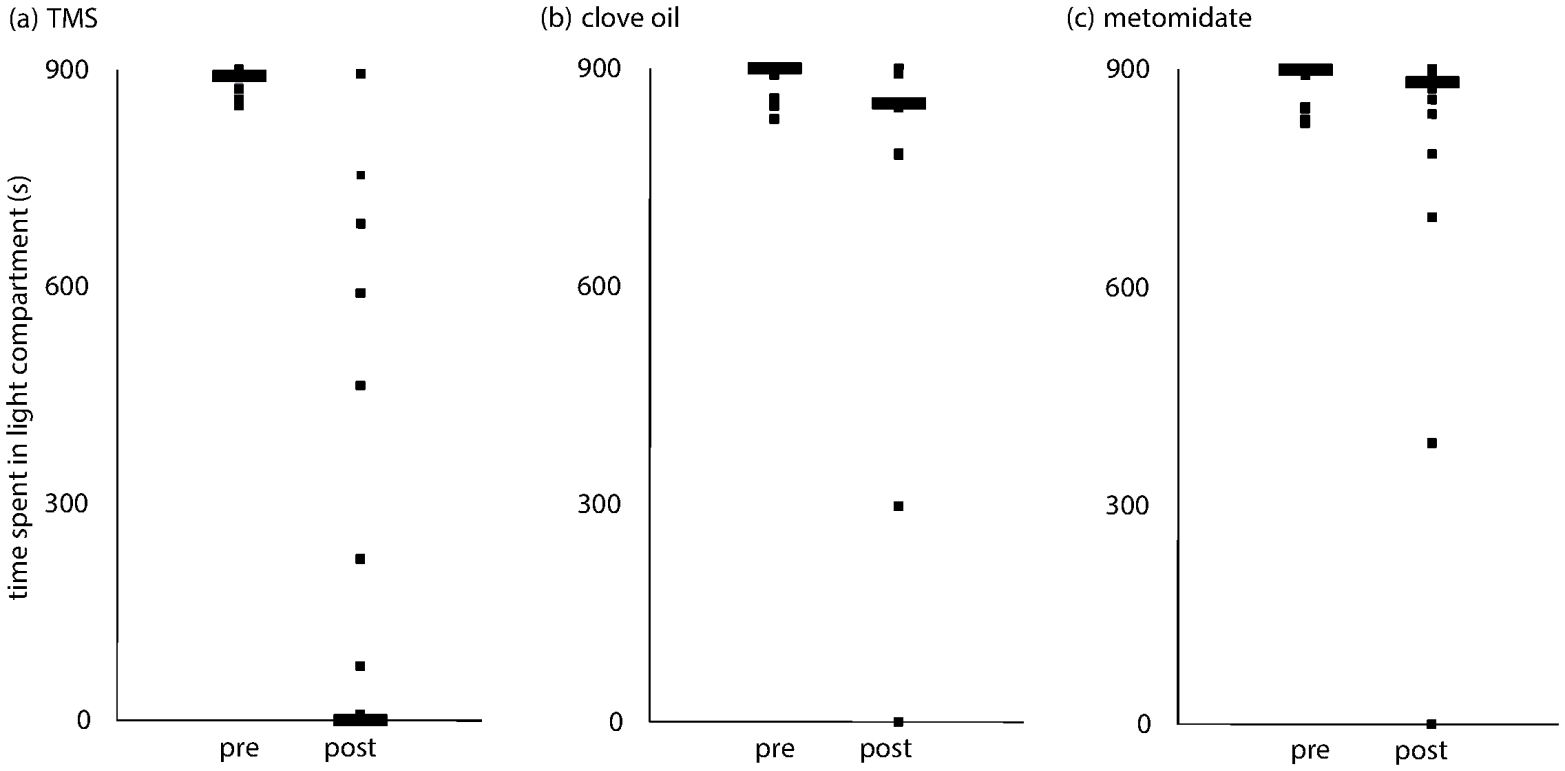
Total time spent in the ‘preferred’ compartment pre- and post- exposure to three euthanasia agents. Total time spent (s) in the light compartment, preferred by all fish before exposure to TMS (a; n = 17), clove oil (b; n = 16), and metomidate hydrochloride (c; n = 18). Times spent are also shown after exposure to these agents. The black bar represents the median. Each square dot shows the value of a single fish. Duplicate values are superimposed.

After exposure testing, all but one of the fish tested with TMS spent less time in the previously preferred light side; fish exposed to both metomidate and clove oil showed less evidence of conditioned place aversion (Chi-square = 14.8, 2 d.f., P<0.001; Figure 3). Nine of 17 fish tested completely rejected the previously preferred side after exposure to TMS; in contrast only 2 of 18 and 3 of 16 showed complete rejection after exposure to metomidate and clove oil, respectively (Table 2, P<0.015).

**Table 2:** Number of fish choosing not to enter the light compartment after exposure to three euthanasia agents.

	Complete Rejection	Re-entered
TMS	9/17	8/17
Clove oil	3/16	13/16
Metomidate hydrochloride	2/18	16/18

Complete rejection was defined as no attempted entries into the previously preferred light compartment. Fish were tested following exposure to TMS (n = 17), clove oil (n = 16), and metomidate hydrochloride (n = 18).

## Discussion

Marking and Meyer's [29] define ideal fish anaesthetics as those that “produce anaesthesia within 3 minutes or less, cause no toxicity to fish at treatment levels, present no mammalian safety problems, leave low tissue residues after a withdrawal time of 1 h or less, and be reasonable in cost”; previous work assessing fish anaesthetics has used these criteria [8]. However, these criteria do not include the capacity of the anaesthetic to cause suffering during induction or recovery. We demonstrate that exposure to TMS causes conditioned place aversion; fish spent less time in their previously preferred side after exposure to TMS than after exposure to the other two chemicals. It is important to note that aversion is displayed when animals are tested in clean tanks. Thus the conditioned place aversion implies that the fish experienced an unpleasant state during induction and learned (in a single exposure) to associate this unpleasant state with the light side of the light/dark apparatus. We found less evidence of conditioned aversion following exposure to metomidate and clove oil, suggesting that these agents are less aversive to zebrafish.

Conditioned place aversion allows the overall experience of the anaesthesia exposure to be taken into account when a fish chooses whether or not to return to the previously preferred compartment. Conditioned place aversion will not be effective if agents have amnesic effects, as the animal will have little memory of the aversive event. The strong conditioned place aversion response to TMS therefore indicates that 1) exposure to this agent is unpleasant for the fish, and 2) that any amnesic effects of TMS are sufficiently mild that the fish are able to associate the environment with this unpleasant experience. The weaker conditioned place aversion to metomidate and clove oil may be because exposure to these agents is less aversive, or because the drugs have stronger amnesic properties.

It is also possible our results reflect an increase in anxiety after exposure to TMS. Fish and other animals tend to hide (or seek a dark environment) when anxious [13], [14]. If fish experienced anxiety following TMS exposure this may explain their changed preference for the dark side of the test apparatus. This effect could be tested by using neutral cues (like striped or coloured sides) in the conditioned place avoidance paradigm and by testing the propensity of fish to seek cover (e.g. under plants) in the home tank.

TMS enters the body via the gills and produces anaesthesia by impeding neuronal signal transmission peripherally to the central nervous system. During the initial phase of anaesthesia using TMS, hyperglycaemia, increased heart rate and respiration are observed, followed by depression of heart rate and ventilation [6]. Anaesthesia using TMS failed to prevent elevation in plasma cortisol during stress-inducing experimental procedures, such as blood sampling [30], [31]. Furthermore, an increase in plasma catecholamine values has been reported in fish exposed to TMS, consistent with a stress response to TMS alone [32], [33].

Readman et al. [34] tested aversion in zebrafish to TMS and other agents using a laminar flow apparatus, and found that that the fish swim to avoid the stream containing TMS when given the option of escaping into untreated water. We had expected similar aversion in the current study and it is not clear why only one fish left the chamber containing the anaesthetic. It is possible that rapid onset of anaesthesia and the narrow diameter of the tube made escape more difficult.

The use of a light/dark box in this study allowed measurement of conditioned place responses, but made it difficult to measure locomotor activities in the dark compartment. We suggest that future studies include assessment of locomotor activities, such as swim speed and direction changes as these could be helpful in assessing responses and depth of anaesthesia.

Metomidate is a non-barbiturate hypnotic that acts on the central nervous system and produces sedation and hypnosis in humans. Used as a clinical and veterinary sedative, it has been shown to inhibit production of cortisol and prevent handling-related glucose responses in fish [23]. Readman et al. [34] also found that zebrafish avoided exposure to etomidate (an analogue to metomidate). Etomidate was not tested in this study, but we found that the anaesthetic experience of metomidate was not sufficiently aversive enough to repel zebrafish from the previously preferred compartment.

Eugenol-based anaesthetics, including AQUI-S and clove oil, are known to inhibit cortisol production and handling-related glucose responses [23]. As the rate of eugenol passage through the gills is dependent on lipid solubility and degree of ionization [35], Keene et al. [30] speculated that eugenol-based anaesthetics are more efficient due to the oils' high solubility, resulting in rapid induction even when using low concentrations of clove oil. Sladky et al. [36] speculates that the oily property of clove oil coats the gill epithelia and consequently prolongs anaesthetic effects. Eugenol has also been reported to have greater effects on the respiratory and cardiac system than TMS, resulting in lower ventilation and heart rates [37]. For euthanasia purposes higher recovery times may be beneficial as a longer buffer time is allowed for secondary euthanasia, reducing the risk of fish recovering.

The unit cost of TMS is lower than metomidate, but the cost of anaesthesia is higher for TMS as the required concentration is higher. Human safety concerns have been expressed for clove oil [38], but we suggest that these risks can be minimized in laboratories with well-trained staff.

We conclude that metomidate and clove oil are humane alternatives to TMS for killing laboratory zebrafish. These anaesthetics may be of use in other fish species, but the diversity in fish physiological responses make it important to assess responses and appropriate concentrations before use.

## Supporting Information

Dataset S1 **The total time spent in the previously preferred side before, during, and after exposure for each fish.**
(XLSX)
